# A polyvalent virosomal influenza vaccine induces broad cellular and humoral immunity in pigs

**DOI:** 10.1186/s12985-023-02153-5

**Published:** 2023-08-16

**Authors:** Vanessa Haach, Ana Paula Almeida Bastos, Danielle Gava, Francisco Noé da Fonseca, Marcos Antônio Zanella Morés, Arlei Coldebella, Ana Cláudia Franco, Rejane Schaefer

**Affiliations:** 1 Laboratório de Virologia, Departamento de Microbiologia, Imunologia e Parasitologia, Instituto de Ciências Básicas da Saúde, Universidade Federal do Rio Grande do Sul, Rua Ramiro Barcelos, 2600, Porto Alegre, Rio Grande Do Sul CEP 90035-003 Brazil; 2 Present Address: Embrapa Sede, Parque Estação Biológica, Brasília, Distrito Federal CEP 70770-901 Brazil; 3 Embrapa Suínos e Aves, BR-153, Km 110, Concórdia, Santa Catarina CEP 89715-899 Brazil

**Keywords:** Influenza A virus, Virosomal vaccine, Humoral immunity, Cellular immunity, Swine

## Abstract

**Background:**

Influenza A virus (IAV) is endemic in pigs globally and co-circulation of genetically and antigenically diverse virus lineages of subtypes H1N1, H1N2 and H3N2 is a challenge for the development of effective vaccines. Virosomes are virus-like particles that mimic virus infection and have proven to be a successful vaccine platform against several animal and human viruses.

**Methods:**

This study evaluated the immunogenicity of a virosome-based influenza vaccine containing the surface glycoproteins of H1N1 pandemic, H1N2 and H3N2 in pigs.

**Results:**

A robust humoral and cellular immune response was induced against the three IAV subtypes in pigs after two vaccine doses. The influenza virosome vaccine elicited hemagglutinin-specific antibodies and virus-neutralizing activity. Furthermore, it induced a significant maturation of macrophages, and proliferation of B lymphocytes, effector and central memory CD4^+^ and CD8^+^ T cells, and CD8^+^ T lymphocytes producing interferon-γ. Also, the vaccine demonstrated potential to confer long-lasting immunity until the market age of pigs and proved to be safe and non-cytotoxic to pigs.

**Conclusions:**

This virosome platform allows flexibility to adjust the vaccine content to reflect the diversity of circulating IAVs in swine in Brazil. The vaccination of pigs may reduce the impact of the disease on swine production and the risk of swine-to-human transmission.

**Supplementary Information:**

The online version contains supplementary material available at 10.1186/s12985-023-02153-5.

## Background

Influenza A virus (IAV) subtypes H1N1, H1N2 and H3N2 are endemic in swine herds globally, causing economic losses for the swine industry and public health concerns. The control of influenza in pigs in Brazil is a challenge due to co-circulation of multiple genetically distinct viruses of subtypes H1N1, H1N2 and H3N2. Human seasonal IAVs of subtypes H1N1 and H1N2 were introduced in swine in the middle of 1980s, and in the early 2000s, respectively, and formed three Brazilian genetic clades within the lineage H1-1B (1B.2.3, 1B.2.4 and 1B.2.6) [1–3]. In the middle 1990s, a human seasonal H3N2 IAV was introduced in swine and diversified into three genetic clades (1990.5.1, 1990.5.2 and 1990.5.3) [3]. Regarding to H1N1 pandemic (pdm) IAV, several human-to-swine spillover events have occurred since 2009, however, only four of these introductions resulted in sustained onward transmission in swine, giving rise to four distinct genetic clusters within the lineage 1A.3.3.2 [4]. A substantial antigenic diversity between distinct subtypes and lineages of Brazilian swine IAVs (swIAVs) has been found, and may impact vaccination [5]. In recent years, the genetic and antigenic diversity of swIAVs has expanded through reassortment events among viruses co-circulating in pigs and accumulation of amino acid changes in genes encoding the viral glycoproteins, hemagglutinin (HA) and neuraminidase (NA) [1, 6]. The HA binds to cell receptors and mediates virus entry into cells, and is the main antigen against which neutralizing antibodies are induced during infection or vaccination [7]. The HA gene is highly variable, harboring mutations that can lead to antigenic variation and, consequently an antigenic mismatch between the vaccine and infection strain that can lead to vaccine failure, and thus immune escape [8].

Vaccination is the most effective measure to mitigate and control influenza-associated morbidity and mortality in swine populations. Additionally, IAV vaccination in pigs contributes to human health by reducing zoonotic transmission and the appearance of "variants" in humans, as well as the emergence of pandemics. However, the rapid viral evolution and co-circulation of multiple distinct IAV lineages pose a challenge for the development of cross-protective vaccines [9]. Whole inactivated influenza virus (WIV) vaccines are commercially available in many countries around the world [10] and induce humoral immune response and protection in pigs against challenge with homologous virus [11]. Live-attenuated influenza vaccine (LAIV) induces a cell-mediated immune response and improved protection against challenge with heterologous virus but may reassort with wild-type IAV or revert virulence [12, 13]. Other vaccine platforms using viral vectors, nucleic acid-based particles and virus-like particles have also been tested in pigs [14–17]. These vaccines stimulate antibody and cell-mediated responses, are safe and can be used to construct polyvalent vaccines that can be updated [18]. In Brazil, a WIV containing the H1N1pdm virus as well as autogenous vaccines have been commercialized since 2014 and 2017, respectively. However, IAV vaccination in pigs is not a common practice in Brazilian farms.

Therefore, novel vaccine strategies that induce wide cross protection, are safe and can be rapidly updated are required. Virosomes consist of reconstituted viral envelopes, but without virus genetic material [19, 20]. Their use is an alternative for the control of influenza in pigs since they mimic virus infection, eliciting a broad immune response [20]. Influenza virosomes preserve the receptor-binding and membrane fusion activities of the HA, allowing the presentation to the major histocompatibility complex (MHC) class I and II, interacting with the immune system through pathways similar to IAVs, and resulting in high immunogenicity [19]. Virosomal influenza vaccines have already been developed for humans and poultry [21, 22]. However, its use in pigs has been poorly studied.

The aim of this study was to assess the immune response kinetics of a virosomal-based influenza vaccine containing the viral envelope proteins of H1N1pdm, H1N2, and H3N2 IAVs in pigs.

## Methods

### Viruses and vaccine

A/swine/Brazil/025-15/2015 1A.3.3.2 (H1N1pdm; NCBI GenBank Accession HA = MH559931 and NA = MH559933; BRMSA 1710), A/swine/Brazil/223-15-1/2015 1B.2.4 (H1N2; NCBI GenBank Accession HA = MH560035 and NA = MH560037; BRMSA 1698) and A/swine/Brazil/028-15-8/2015 1990.5.2 (H3N2; NCBI GenBank Accession HA = MH559963 and NA = MH559965; BRMSA 1697) were the viruses used in this study. H1N1pdm and H1N2 viruses were propagated in specific pathogen-free (SPF) embryonated chicken eggs and H3N2 virus was propagated in Madin–Darby canine kidney (MDCK) cells [23]. The three viruses were individually concentrated by tangential ultrafiltration, followed by ultracentrifugation, and the pellets were resuspended in TNE buffer (10 mM Tris, 100 mM NaCl and 1 mM EDTA, pH 7.4).

The virosomal influenza vaccine was prepared as previously described by Fonseca et al. [24]. Briefly, the three concentrated viruses were mixed with 200 mM of 1,2-dicaproyl-sn-glycero-3-phosphocholine (DCPC), then diluted 1:2 (v/v) in TNE buffer, and the final mixture was incubated in an ice bath for 30 min to ensure viral dissolution. Viral nucleocapsids were removed by ultracentrifugation (100,000 × g for 30 min at 4 °C). The supernatant was extensively dialyzed against TNE buffer for 48 h at 4 °C to remove the DCPC, which led to the self-assembling of virosomes. The virosomal particle was characterized previously by Fonseca et al. [24]. The influenza vaccine contained 128 µg of total HA and 20% (v/v) of Emulsigen®-D (MVP Laboratories) per mL. The HA concentration corresponded to 6, 21 and 73% for H1N1, H1N2 and H3N2, respectively, as evaluated by SDS-PAGE [24].

### Animal study design

**Fig. 1:**
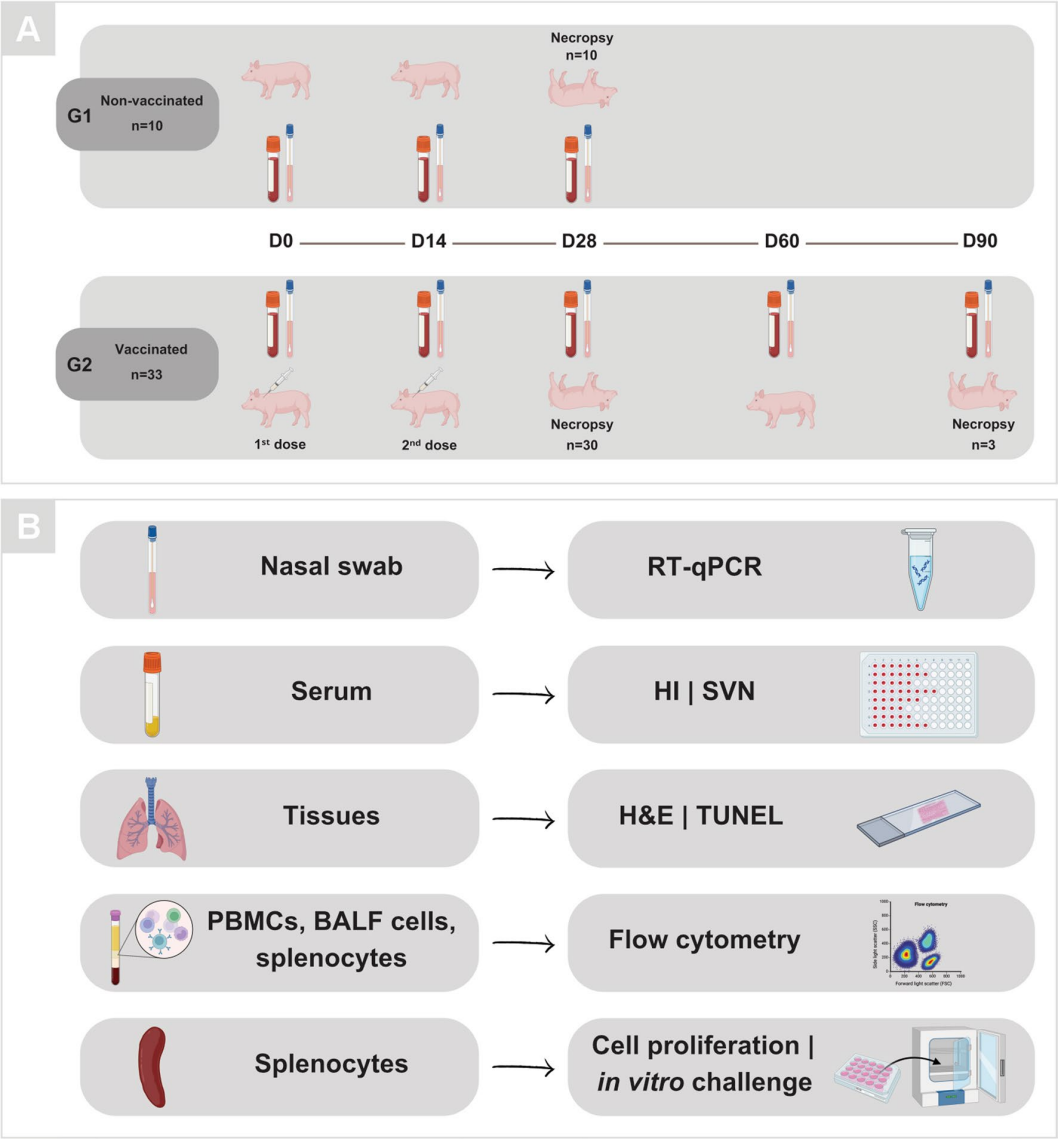
Experimental design. **A** Timeline of G1 (non-vaccinated) and G2 (vaccinated) groups, highlighting the time points (D0, D14, D28, D60 and D90) of blood and nasal swab collection, administration of virosomal influenza vaccine (first and second dose) and necropsy of pigs. **B** Main laboratory assays carried out with biological samples collected from pigs of G1 and G2 groups. BALF = Bronchoalveolar lavage fluid; D = day; H&E = hematoxylin and eosin; HI = hemagglutination inhibition; PBMCs = peripheral blood mononuclear cells; SVN = serum virus neutralization. Illustration created with BioRender.com

Forty-three (43) four-week-old pigs were obtained from a SPF herd composed of crossbreed pigs (MS 115–composite terminal sire developed by Embrapa Swine and Poultry with Landrace × Large White sows). All pigs were previously tested negative for IAV antibodies (Multispecies Influenza A Antibody Test kit, BioChek) and IAV RNA [25]. All pigs were transferred to the biosafety level 1 (BSL1) experimental facility one week before the beginning of the experiment and cared for in compliance with the Animal Use Ethics Committee of Embrapa Swine and Poultry (protocol number 001/2017). The pigs were randomly divided into two groups: G1: 10 non-vaccinated pigs; and G2: 33 vaccinated pigs. Pigs from G2 were vaccinated on days (D) 0 and 14 with 1 mL of the adjuvanted virosomal IAV vaccine, by the intramuscular route in the neck. Pigs were daily monitored for clinical signs, behavior, appetite and temperature or any adverse effects related to vaccination. Blood and nasal swab samples were collected from all pigs on D0, D14 and D28. Three pigs from the G2 group were kept in the experimental facility until D90 to assess the long-term immunity induced by vaccination, and additional blood and nasal swab samples were collected on D60 and D90. The pigs were anesthetized with 6 mg/kg of Zoletil® (Zolazepam + Tiletamine; 100 mg/mL, Virbac) by the intramuscular route and euthanized with one step electrocution followed by bleeding. Necropsy was performed on D28 for the G1 and G2 groups, and on D90 for the three remaining pigs from the G2 group. Spleen from all pigs were excised and kept in RPMI 1640 medium supplemented with 1 × penicillin, streptomycin and fungizone for the in vitro cell proliferation assay. Bronchoalveolar lavage fluid (BALF) [26] and blood with anticoagulant (BD Vacutainer® EDTA K2) were collected for cell profile analysis by flow cytometry. Lung, mediastinal lymph node, spleen, liver and kidney fragments were collected and preserved in 4% paraformaldehyde for histopathological assessment (Fig. 1A, B).

### Diagnostic microbiology

The presence of other respiratory pathogens was investigated as follows: RNA was extracted from nasal swab samples using MagMAX™ Viral RNA Isolation kit (Thermo Fisher Scientific) and tested by RT-qPCR targeting IAV/matrix gene [25]. Serum and BALF samples were submitted for DNA extraction using the DNeasy® Blood & Tissue kit (Qiagen). Serum samples were evaluated by qPCR for detection of porcine circovirus type 2 (PCV2) [27]. BALF samples were evaluated by qPCRs for detection of *Actinobacillus pleuropneumoniae* [28], *Glaesserella parasuis* [29], *Mycoplasma hyopneumoniae* [30] and *Pasteurella multocida* [31]. Samples were not tested for porcine reproductive and respiratory syndrome virus (PRRSV), since Brazil is considered free of PRRS [32].

### Histopathological evaluation and immunohistochemistry

Lung, mediastinal lymph node, spleen, liver and kidney tissue samples fixed in 4% paraformaldehyde, were routinely processed and stained with hematoxylin and eosin (H&E) [33]. The mediastinal lymph node samples were evaluated for PCV2 detection by immunohistochemistry [34]. Additionally, to confirm that the vaccine was not cytotoxic to the pigs, the qualitative and quantitative analysis of apoptotic cells was performed in lung, mediastinal lymph node, spleen, liver and kidney tissues by using the DeadEnd™ Colorimetric TUNEL (Terminal deoxynucleotidyl transferase-mediated deoxyuridine triphosphate nick-end labeling) System kit (Promega) in accordance with the manufacturer's recommendations but using the 3-amino-9-ethylcarbazole (AEC) chromogen. Microscopic analysis was carried out using optical microscopy (Axio Scope.A1, Zeiss) at 400 × magnification and consisted of blind quantification of positive and total cells in ten different fields, each field measuring 37,000 µm^2^.

### Serological analysis

Serum samples were evaluated for HA-specific and virus-neutralizing antibodies. For this, the serum samples were treated and submitted to the hemagglutination inhibition (HI) [35] and serum virus neutralization (SVN) [36] assays. The same IAV strains composed the virosomal vaccine in this study were used as antigens in the HI and SVN assays: A/swine/Brazil/025-15/2015(H1N1), A/swine/Brazil/223–15-1/2015(H1N2) and A/swine/Brazil/028-15-8/2015(H3N2). The results were reported as geometric mean antibody titers.

### Determination of cellular profile

The cellular profile from the blood and BALF collected from the pigs was assessed by flow cytometry. Heparinized whole blood samples were diluted 1:3 (v/v) in PBS and the peripheral blood mononuclear cells (PBMCs) were isolated by density gradient centrifugation (Ficoll-Paque™ PLUS, GE Healthcare), following the manufacturer’s recommendations. PBMCs were resuspended in flow cytometry buffer (PBS supplemented with fetal bovine serum (FBS, 2% v/v, Gibco), bovine serum albumin (BSA, 2% w/v, Sigma-Aldrich) and sodium azide (0.01% w/v, Sigma-Aldrich)). BALF samples were centrifuged at 800 × g for 10 min at 4 °C and the pellet was resuspended in flow cytometry buffer. The PBMC and BALF cell concentration was adjusted and distributed to approximately 1 × 10^6^ cells/well (96-well plate). The cells were stained with specific monoclonal antibodies (mAbs) [37] to assess major lymphocyte, monocyte/macrophage, granulocyte and natural killer (NK) cell populations by four-color flow cytometry (Table 1). The PBMCs and BALF cells resuspended in flow cytometry buffer were incubated for 30 min at room temperature with a cocktail of specific mAbs. The fluorochrome-labeled mAbs used for the PBMCs and BALF cells are described in Table 2. For intracellular staining, the cells were treated with the Cytofix/Cytoperm™ Fixation/Permeabilization kit (BD), according to the manufacturer's instructions, and then the cells were stained with CD79a (the epitope recognized by the mAb is located in the cytoplasmic domain) and CD3 (the PPT3 clone recognizes an extracellular and intracellular epitope on CD3) mAbs. All antibodies used in the staining were previously titrated for their optimum concentrations. To evaluate fluorochrome unspecific staining, respective isotype controls for anti-IgG1, anti-IgG2a and anti-IgG2b were analyzed in the preliminary procedure to set up technical parameters.

**Table 1:** Antibodies used in flow cytometry

Antibody	Species	Clone	Isotype	Fluorochrome	Dilution
7-AAD	NA	NA	NA	7-AAD	1:400
Granulocytes	Mouse anti-pig	6D10	IgG2a	FITC	Neat
Macrophages	Mouse anti-pig	BA4D5	IgG2b	RPE^a^	Neat
CSF1R	Mouse anti-pig	ROS8G11	IgG2a	APC	Neat
SLA Class II	Mouse anti-pig	2E9/13	IgG2b	FITC	Neat
IFN-γ	Mouse anti-pig	P2G10	IgG1	PerCP-Cy5.5	Neat
CD3e	Mouse anti-pig	PPT3	IgG1	FITC	1:10
				RPE-Cy7^b^	
				APC^c^	
CD4α	Mouse anti-pig	MIL17	IgG2b	RPE	1:100
CD5	Mouse anti-pig	1H6/8	IgG2a	FITC	Neat
CD8α	Mouse anti-pig	MIL12	IgG2a	FITC	1:100
				RPE	
CD14	Mouse anti-pig	MIL2	IgG2b	FITC	1:10
CD16	Mouse anti-pig	G7	IgG1	RPE	1:10
SWC7 or CD19	Mouse anti-bovine	CC55	IgG1	RPE-Cy7^b^	Neat
CD25	Mouse anti-pig	K231.3B2	IgG1	RPE-Cy7^b^	Neat
CD27 or SWC2	Mouse anti-pig	B30C7	IgG1	APC	1:10
CD45RA	Mouse anti-pig	MIL13	IgG1	FITC	Neat
CD79a	Mouse anti-human	HM57	IgG1	RPE	1:10
CD335	Mouse anti-pig	VIV-KM1	IgG1	APC	1:100
IgG1 isotype control	Mouse	NA	IgG1	FITC	1:10
				RPE	
				APC	
	Mouse anti-pig			RPE-Cy7^b^	Neat
				PerCP-Cy5.5^d^	Neat
IgG2a isotype control	Mouse	NA	IgG2a	FITC	1:10
				RPE	
				APC	
IgG2b isotype control	Mouse	NA	IgG2b	FITC	1:10
				RPE	

^a^Monoclonal antibody dilution following RPE labeling (Serotec)

^b^Monoclonal antibody dilution following RPE-Cy7 labeling (Serotec)

^c^Monoclonal antibody dilution following APC labeling (Serotec)

^d^Monoclonal antibody dilution following PerCP-Cy5.5 labeling (Serotec)

NA = Not applicable

**Table 2:** Panels of fluorochrome-labeled monoclonal antibodies

Panel	Fluorochrome-labeled monoclonal antibodies for PBMCs
A	7-AAD (BD Biosciences)
B	RPE-macrophages (clone BA4D5), FITC-SLAII (clone 2E9/13)
C	FITC-CD14 (clone MIL2), RPE-CD16 (clone G7)
D	FITC-CD3e (clone PPT3), RPE-CD4α (clone MIL17), RPE-Cy7-CD25 (clone K231.3B2)
E	RPE-Cy7-CD3e (clone PPT3), RPE-CD4α (clone MIL17), FITC-CD8α (clone MIL12), APC-CD335 (clone VIV-KM1)
F	RPE-Cy7-CD3e (clone PPT3), RPE-CD4α (clone MIL17), APC-CD27 (or SWC2, clone B30C7), FITC-CD45RA (clone MIL13)
G	RPE-CD79a (clone HM57), RPE-Cy7-SWC7 (or CD19, clone CC55), FITC-CD5 (clone 1H6/8)
Panel	Fluorochrome-labeled monoclonal antibodies for BALF cells
A	7-AAD (BD Biosciences)
B	FITC-granulocytes (clone 6D10)
C	APC-CSF1R (clone ROS8G11), FITC-SLAII (clone 2E9/13)
D	FITC-CD14 (clone MIL2), RPE-CD16 (clone G7)
E	RPE-Cy7-CD3e (clone PPT3), RPE-CD79a (clone HM57), FITC-CD5 (clone 1H6/8)
F	RPE-Cy7-CD3e (clone PPT3), RPE-CD4α (clone MIL17), FITC-CD8α (clone MIL12), APC-CD335 (clone VIV-KM1)
Panel	Fluorochrome-labeled monoclonal antibodies for in vitro cell proliferation assay
A	7-AAD (BD Biosciences), RPE-macrophages (clone BA4D5)
B	RPE-CD79a (clone HM57), RPE-Cy7-SWC7 (or CD19, clone CC55)
C	APC-CD3e (clone PPT3), RPE-CD4α (clone MIL17), RPE-Cy7-CD25 (clone K231.3B2)
D	APC-CD3e (clone PPT3), RPE-CD8α (clone MIL12), RPE-Cy7-CD25 (clone K231.3B2)
E	RPE-Cy7-CD3e (clone PPT3), RPE-CD4α (clone MIL17), APC-CD27 (or SWC2, clone B30C7)
F	RPE-Cy7-CD3e (clone PPT3), RPE-CD8α (clone MIL12), APC-CD27 (or SWC2, clone B30C7)
G	APC-CD3 (clone PPT3), RPE-CD8α (clone MIL12), PerCP-Cy5.5-IFN-γ (clone P2G10)

Monoclonal antibodies used to stain peripheral blood mononuclear cells (PBMCs), bronchoalveolar lavage fluid (BALF) cells and in vitro cell proliferation assay

The stained cells were acquired using an Accuri™ C6 Plus flow cytometer (BD). Fifty thousand events were analyzed based on forward scatter (FSC) and side scatter (SSC), using Accuri™ C6 Plus (BD) and FlowJo™ (Tree Star Inc.) software. Before sample analysis, flow cytometer settings were verified using Cytometer Setup and Tracking beads (CS&T beads, BD) as described in the manufacturer's instructions. Compensation beads were used with single stains of each antibody to establish the compensation settings. The SSC threshold was set at 8,000 units to eliminate debris. Gates considered to indicate positive and negative staining cells were set based on fluorescence minus one (FMO) tests of samples, and these gates were performed systematically on each sample, allowing minor adjustments for SSC variability. Dead cells were excluded by discrimination with 7-AAD dye, according to our protocol previously described [38].

### In vitro cell proliferation assay

Due to the lack of an adequate biosafety structure for the challenge of pigs with influenza virus, the in vitro challenge by culturing swine splenocytes and stimulating them with the three vaccine virus strains was performed. For this, spleen fragments were mechanically dissociated under aseptic conditions and filtered through a 70 μm Nylon Cell Strainer (Corning). After this, red blood cells were depleted using Pharm Lyse™ Buffer (BD Biosciences) for five minutes at room temperature. The splenocytes were obtained after the addition of RPMI 1640 medium to stop the lysis reaction, followed by washing the cells twice with RPMI 1640 medium. Splenocytes were labeled with 2.5 µM carboxyfluorescein succinimidyl ester (CFSE) by applying the CellTrace™ CFSE Cell Proliferation kit (Invitrogen), for 15 min at 37 °C in the dark. The reaction was stopped by adding six volumes of RPMI 1640, supplemented with 10% FBS, followed by incubation for 5 min in an ice bath, in the dark. Finally, the cells were washed three times with RPMI 1640-10% FBS, and further resuspended at a concentration of 5 × 10^6^ cells/mL in the same medium. Viable cells were cultured in RPMI 1640 medium supplemented with 10% FBS (Gibco), 1 mM GlutaMAX (Gibco), 25 mM HEPES (Sigma-Aldrich), 1 mM sodium pyruvate (Sigma-Aldrich), 50 M 2-mercaptoethanol (Gibco) and 100 U/mL penicillin–streptomycin (Sigma-Aldrich) at 37 °C under 5% CO_2_. During 96 h in the dark, cells were stimulated in vitro by adding 8000 TCID_50_/mL of each of the three vaccine viruses separately (H1N1, H1N2 and H3N2). For the negative control, culture medium was added to one well of the microplate (non-virus-stimulated cells). For the positive control, two wells of the microplate were prepared, one well with 3 µg/mL of Concanavalin A from *Canavalia ensiformis* (Sigma-Aldrich) and another well with 10 µg/mL of lipopolysaccharide from *Escherichia coli* (Sigma-Aldrich).

CFSE in combination with mAbs, enabled the concomitant access to cell proliferation and activation status of cell subpopulations. Flow cytometry analysis was performed to identify and quantify lymphocyte subpopulations (CD3, CD4, CD8, CD79a mAbs), to measure the levels of cellular activation marker expression (CD25, CD19 mAbs), cellular memory marker expression (CD27 mAb), interferon-γ (IFN-γ) cytokine and also to quantify macrophages (BioRad Serotec) (Table 1). Proliferation was detected by loss of CFSE fluorescence [38].

Cells obtained after 96 h of stimulation were resuspended in flow cytometry buffer, and the cell concentration was adjusted and distributed to approximately 1 × 10^5^ cells/well (96-well plate). To assess major lymphocyte and macrophage populations, cells were stained with the fluorochrome-labeled mAbs described in Table 2. For intracellular staining, the cells were treated with the Cytofix/Cytoperm™ Fixation/Permeabilization kit (BD), according to the manufacturer’s instructions, and stained with CD79a, CD3 and IFN-γ mAbs. The respective isotype controls and the acquiring stained cells were performed as described in the previous section.

**Fig. 2:**
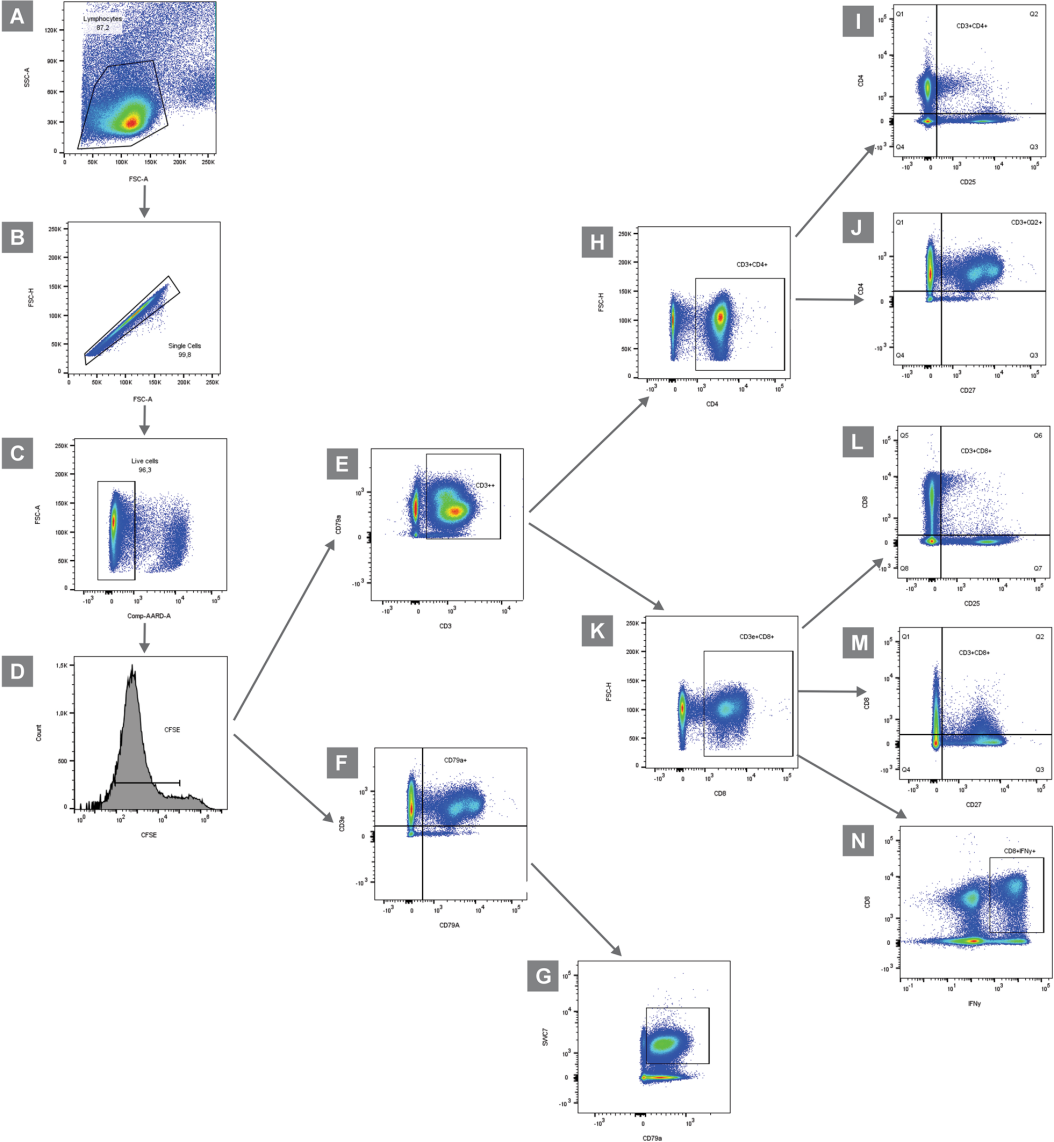
Hierarchical gating strategy applied to samples from the experiment. **A** Flow cytometry dot plot gate shows lymphocytes (left). **B** The forward scatter (FSC) A × FSC-H dispersion was used to gate singlet cells. **C** Viable cells were defined by a gate encompassing the 7-AAD negative cells in a FSC-A vs. 7-AAD dot-plot. **D** Flow cytometry histograms gated into CFSE^+^ events displaying the CFSE^low^ region (the cell proliferation region). Results of cell proliferation with H1N1, H1N2 and H3N2 challenges. **E** From the viable cell gate (7-AAD negative), the CD3 vs. CD79a negative dot-plot was used to define T lymphocytes, and **F** CD79a positive vs. CD3 negative dot-plot was used to define B lymphocytes. **G** SWC7 vs. CD79a from a gate on cell population, the frequencies of conventional B cells. **H**, **K** From a gate on each cell population (exemplified here by CD4^+^ T cells and CD8^+^ T cells, respectively), the frequencies of **I** effector CD4^+^ T cells (CD4^+^CD25^+^), **J** central memory CD4^+^ T cells (CD4^+^CD27^+^), **L** effector CD8^+^ T cells (CD8α^+^CD25^+^), **M** central memory CD8^+^ T cells (CD8α^+^CD27^+^), and **N** cytotoxic CD8^+^ T cells (CD8α^+^IFNγ^+^) were determined in the respective dot-plots

The gate was performed as described in a previous study conducted by our research group [38]. Gates were set using the non-virus-stimulated sample for each individual pig. In summary, the gate was based on forward scatter (FSC) and side scatter (SSC) properties to estimate lymphocyte population and debris exclusion. The doublet cells were subjected to doublet plotting showing forward scatter height (FSC-H) against forward scatter area (FSC-A). Dead cells were excluded from the analysis using 7-AAD staining. Counterstaining with CD3e/CD79a allowed us to gate on T and B cells, respectively (Fig. 2). Among T lymphocytes, subsets of CD4^+^ and CD8^+^ T cells were captured using panels. The gate to identify monocyte/macrophage cell population was based on which T lymphocytes could be recovered from CD4^bright^ cells. Among B-lymphocytes cells were defined as the sum of CD79a^+^, the gate is similar to what has been described for T lymphocytes. The gate for positive cells to CD79a^+^ was determined, which was differentiated in SWC7^+^ (conventional B cells).

### Statistical analysis

Differences between vaccinated (G2) and non-vaccinated (G1) groups were evaluated using the two-sided Student's t test in the statistical analysis system (SAS) [39]. *P* values ≤ 0.05 were considered statistically significant.

## Results

### Absence of clinical signs and respiratory pathogens

No respiratory clinical signs, changes in behavior, appetite or hyperthermia were observed in the pigs during the experiment. Also, no local inflammatory reactions or any adverse effects related to the vaccination were identified. During the experiment, two pigs died, one on D14 (from G2 group) and another on D22 (from G1 group). Both deaths were not related to the experiment. The pig from G2 group had unintended lesion on vagus nerve during blood collection on jugular groove which led to respiratory and cardiac depression followed by death. For the pig from G1 group, a necropsy was performed and no macroscopic or microscopic lesions were observed. Thus, the G1 and G2 groups remained with 9 pigs and 32 pigs, respectively.

All nasal swab samples collected on D0, D14, D28, D60 and D90 were negative for IAV by RT-qPCR. Moreover, serum samples collected on D0, D28 and D90 were negative for PCV2 and BALF samples were negative for *Actinobacillus pleuropneumoniae*, *Glaesserella parasuis*, *Mycoplasma hyopneumoniae* and *Pasteurella multocida* by qPCR.

### Safety of the virosomal influenza vaccine in pigs

In the histopathological analysis, no significant lesions were detected in lung, spleen, liver and kidney tissue samples collected from all pigs. However, in mediastinal lymph node samples, follicular lymphoid hyperplasia was observed in 71.87% (23/32) of pigs from the G2 group, and mild histiocytic infiltration was observed in 11.11% (1/9) of pigs from the G1 group and 15.62% (5/32) from the G2 group. All mediastinal lymph node samples tested negative for PCV2 by immunohistochemistry.

In the TUNEL assay, there was no difference in the number of apoptotic cells observed in lung, mediastinal lymph node, spleen, liver and kidney tissues between non-vaccinated pigs (G1 group) and vaccinated pigs (G2 group) (Additional file 1: Fig. S1).

The polyvalent influenza virosome vaccine proved to be safe and non-cytotoxic to pigs, as demonstrated by the results obtained in the TUNEL assay and by the lack of adverse reactions after vaccination. The follicular lymphoid hyperplasia observed in the mediastinal lymph nodes from vaccinated pigs remains to be investigated, since immunohistochemistry of mediastinal lymph nodes and qPCR from serum samples were negative to PCV2.

### Virosomal influenza vaccine elicited humoral immune response in pigs

All serum samples from the non-vaccinated pigs (G1 group) were negative for IAV by HI and SVN assays.

**Fig. 3:**
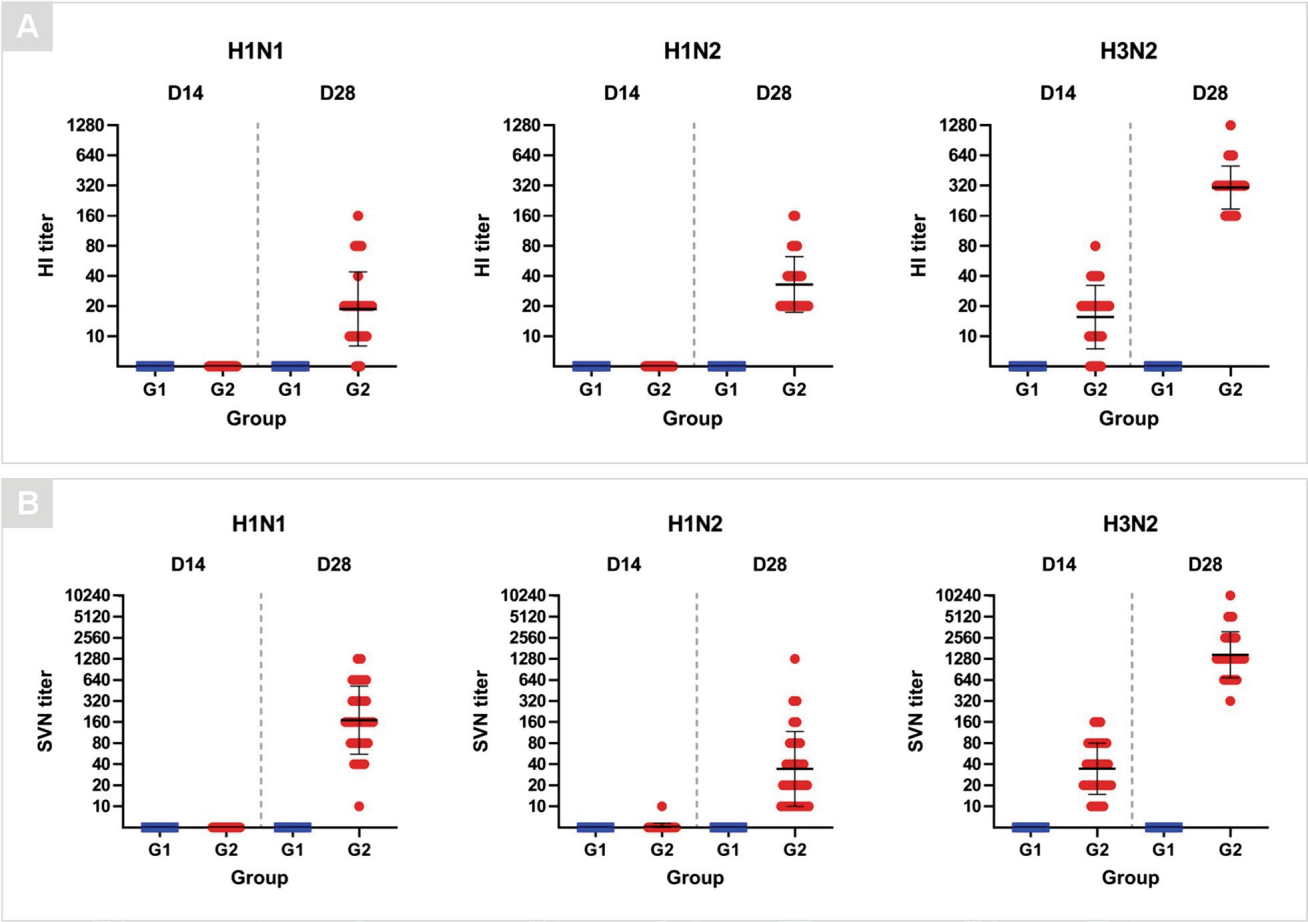
Hemagglutination inhibition (HI) and serum virus neutralization (SVN) assays. Antibody titers by **A** HI and **B** SVN assays for H1N1, H1N2 and H3N2 subtypes of serum samples collected from pigs in the non-vaccinated (G1) and vaccinated (G2) groups on D14 and D28 post-vaccination. Data are shown for each pig per group and the black lines represent the geometric mean titers ± standard deviation

For the vaccinated pigs (G2 group), antibodies to H3N2 virus were detected in 18.75% (6/32) of the pigs (HI titers of 40–80) 14 days after the first vaccine dose. Antibodies to the three vaccine antigens were detected after the second vaccine dose (D28) as follows: for H1N1 virus, 18.75% (6/32) of pigs had HI titers of 40–160; for H1N2 virus, 46.88% (15/32) of pigs had HI titers of 40–160; and for H3N2 virus, 100% (32/32) of pigs had HI titers of 160–1280 (Fig. 3A). Antibodies to H1N2 (HI titer of 40) and H3N2 (HI titer of 160) were detected in one out of three pigs from the G2 group on D90 (Additional file 2: Fig. S2A).

Virus-neutralizing-antibodies for H1N2 were detected in 3.13% (1/32) of pigs (titer of 10) and for H3N2 in 100% (32/32) of pigs (titers of 10–160) 14 days after the first vaccine dose. On D28, virus-neutralizing antibodies were detected in all pigs from the G2 group as follows: antibodies titers ranged from 10 to 1280 for H1N1 and H1N2 viruses, and ranged from 320 to 10,240 for H3N2 virus (Fig. 3B). On D90, one out of three pigs from G2 group had antibodies for H1N1 (titer of 320) and H1N2 (titer of 20), and all three pigs had antibodies for H3N2 (titers ranging from 40 to 320) (Additional file 2: Fig. S2B).

### Virosomal influenza vaccine elicited cellular immune response in pigs

#### *Significant induction of cell proliferation in vaccinated pigs after *in vitro* stimulation*

**Fig. 4:**
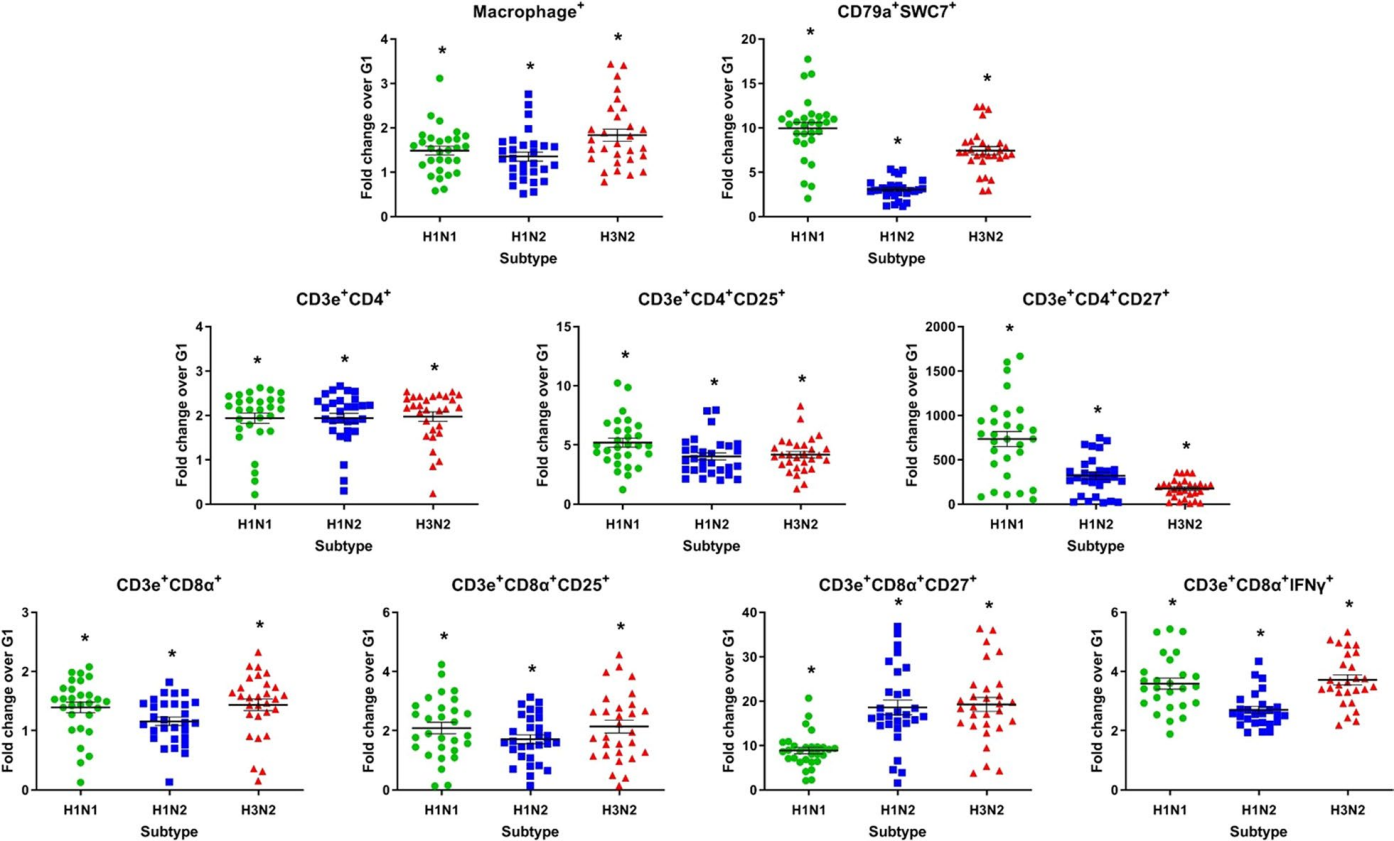
In vitro cell proliferation assay. Immune cells in the splenocyte proliferation assay stimulated with the vaccine viruses (H1N1, H1N2 and H3N2) were compared as a fold change from the vaccinated group (G2) to the non-vaccinated group (G1) on D28 post-vaccination. Data are shown for each pig per virus subtype, and the black lines represent the mean ± standard error. Asterisks (*) denote significant differences between non-vaccinated (G1) and vaccinated (G2) groups (*P* ≤ 0.05)

Nine different cell subsets were defined in the in vitro stimulated splenocyte proliferation assay: macrophage^+^ (monocytes/macrophages), CD79a^+^SWC7^+^ (B lymphocytes), CD3e^+^CD4^+^ (CD4^+^ T lymphocytes), CD3e^+^CD4^+^CD25^+^ (effector CD4^+^ T cells), CD3e^+^CD4^+^CD27^+^ (central memory CD4^+^ T cells), CD3e^+^CD8α^+^ (CD8^+^ T lymphocytes), CD3e^+^CD8α^+^CD25^+^ (effector CD8^+^ T cells), CD3e^+^CD8α^+^CD27^+^ (central memory CD8^+^ T cells) and CD3e^+^CD8α^+^IFNγ^+^ (cytotoxic T lymphocytes producing IFN-γ). In all cell subsets evaluated for the three vaccine viruses (H1N1, H1N2 and H3N2), higher cell counts were observed in the G2 group on D28 compared to the G1 group (*P* ≤ 0.05) (Fig. 4; Additional file 3: Table S1).

A high level of cellular proliferation was still detected in pigs from the G2 group, three months after the first vaccine dose. Furthermore, on D90, CD79a^+^SWC7^+^, CD3e^+^CD4^+^CD27^+^ and CD3e^+^CD8α^+^IFNγ^+^ cell subsets for H1N1, H1N2 and H3N2; CD3e^+^CD8α^+^CD27^+^ cell subset for H1N1 and H3N2; CD3e^+^CD4^+^CD25^+^ cell subset for H1N2; and macrophage^+^ cell subset for H3N2 were statistically significant (*P* ≤ 0.05) (Additional file 4: Fig. S3A; Additional file 3: Table S1).

#### Cellular profile in vaccinated pigs

**Fig. 5:**
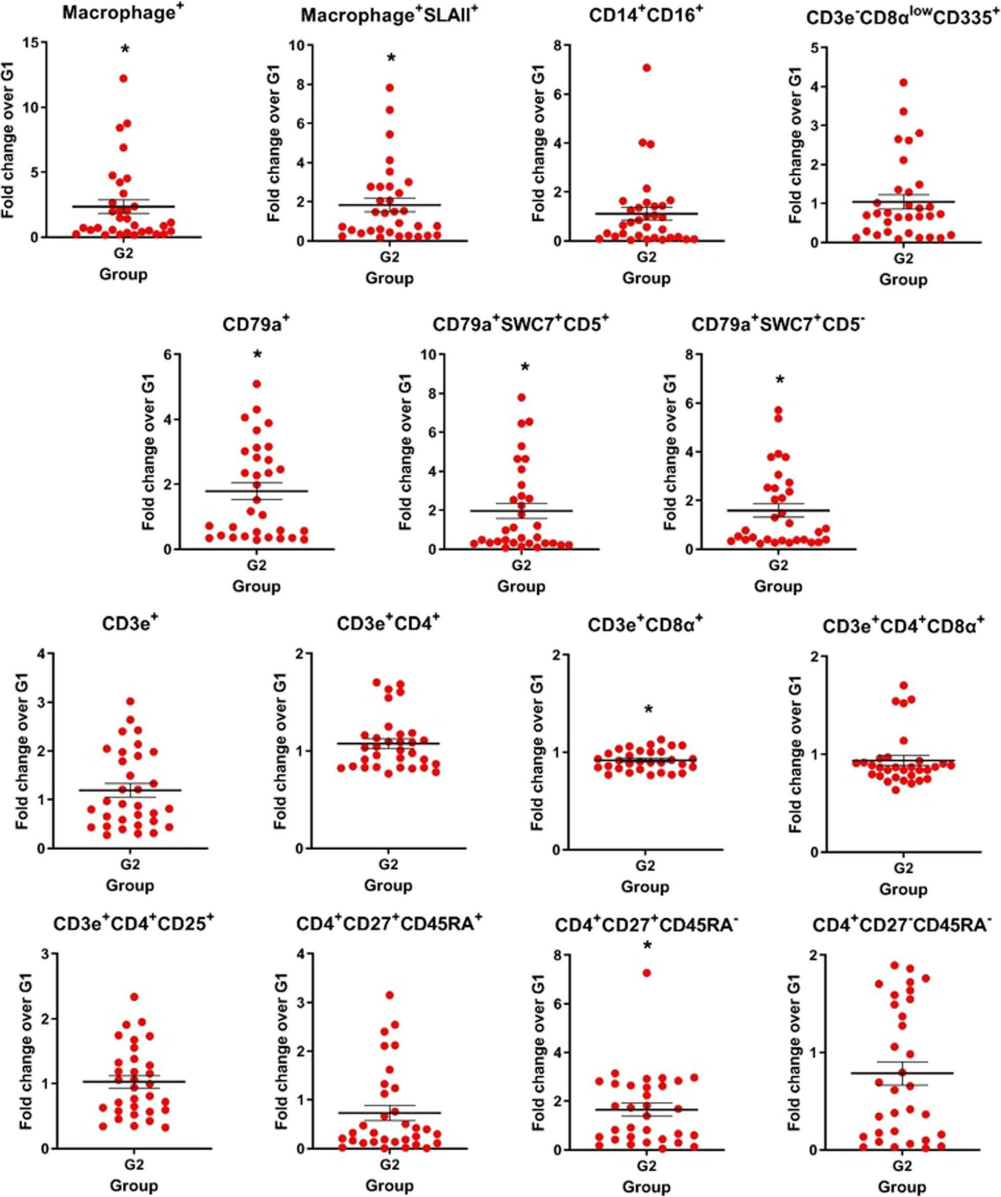
Cell profile in blood. Immune cells in the peripheral blood mononuclear cells (PBMCs), as a fold change from the vaccinated group (G2) over the non-vaccinated group (G1) on D28 post-vaccination. Data are shown for each pig and the black lines represent the mean ± standard error. Asterisks (*) denote significant differences between non-vaccinated (G1) and vaccinated (G2) groups (*P* ≤ 0.05)

Fifteen different cell subsets were defined in the PBMCs: macrophage^+^ (macrophages), macrophage^+^SLAII^+^ (active macrophages), CD14^+^CD16^+^ (monocytes/macrophages), CD3e^−^CD8α^low^CD335^+^ (natural killer cells), CD79a^+^ (total B lymphocytes), CD79a^+^SWC7^+^CD5^+^ and CD79a^+^SWC7^+^CD5^−^ (conventional B cells), CD3e^+^ (total T lymphocytes), CD3e^+^CD4^+^ (CD4^+^ T lymphocytes), CD3e^+^CD8α^+^ (CD8^+^ T lymphocytes), CD3e^+^CD4^+^CD8α^+^ (CD4^+^CD8^+^ double-positive cells), CD3e^+^CD4^+^CD25^+^ (effector CD4^+^ T cells), CD4^+^CD27^+^CD45RA^+^ (naive CD4^+^ T cells), CD4^+^CD27^+^CD45RA^−^ (central memory CD4^+^ T cells), and CD4^+^CD27^−^CD45RA^−^ (effector memory CD4^+^ T cells). A high cell count of macrophage^+^, macrophage^+^SLAII^+^, CD79a^+^, CD79a^+^SWC7^+^CD5^+^, CD79a^+^SWC7^+^CD5^−^, CD3e^+^CD8α^+^ and CD4^+^CD27^+^CD45RA^−^ was detected in the G2 group on D28 compared to the G1 group (*P* ≤ 0.05) (Fig. 5). Moreover, on D90, macrophage^+^, macrophage^+^SLAII^+^, CD14^+^CD16^+^ and CD3e^+^ cell subsets were significantly higher (*P* ≤ 0.05) (Additional file 4: Fig. S3B).

**Fig. 6:**
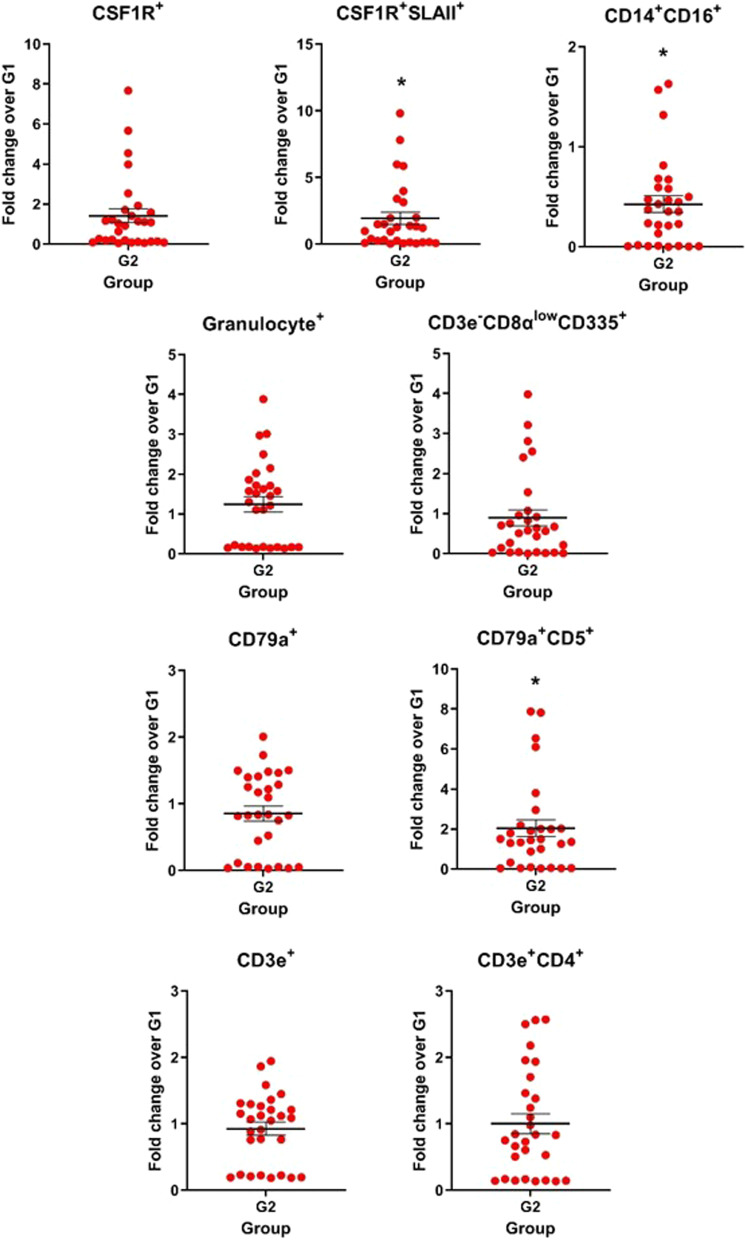
Cell profile in bronchoalveolar lavage fluid (BALF). Immune cells in the BALF cells, as a fold change from the vaccinated group (G2) over the non-vaccinated group (G1) on D28 post-vaccination. Data are shown for each pig and the black lines represent the mean ± standard error. Asterisks (*) denote significant differences between non-vaccinated (G1) and vaccinated (G2) groups (*P* ≤ 0.05)

Nine different cell subsets were defined in the BALF cells: CSF1R^+^ (alveolar macrophages), CSF1R^+^SLAII^+^ (active alveolar macrophages), CD14^+^CD16^+^ (monocytes/macrophages), granulocyte^+^ (granulocytes), CD3e^−^CD8α^low^CD335^+^ (natural killer cells), CD79a^+^ (total B lymphocytes), CD79a^+^CD5^+^ (conventional B cells), CD3e^+^ (total T lymphocytes) and CD3e^+^CD4^+^ (CD4^+^ T lymphocytes). A high cell count of CSF1R^+^SLAII^+^, CD14^+^CD16^+^ and CD79a^+^CD5^+^ was observed in the G2 group on D28 compared to the G1 group (*P* ≤ 0.05) (Fig. 6). In addition, on D90, CSF1R^+^, CSF1R^+^SLAII^+^, CD14^+^CD16^+^ and CD3e^−^CD8α^low^CD335^+^ cell subsets were significantly higher (*P* ≤ 0.05) (Additional file 4: Fig. S3C).

## Discussion

The genetic and antigenic diversity of swIAVs in Brazil has increased since the emergence of H1N1pdm in 2009 [2, 40–43]. Currently, distinct lineages of H1N1, H1N2 and H3N2 IAVs circulate in swine herds across several Brazilian states [2, 3]. In addition, the Brazilian swIAVs are genetically distinct from the viruses circulating in pigs in other countries [1–3]. Consequently, the development of influenza vaccines that incorporate this genetic and antigenic diversity and can rapidly be updated, including locally adapted swIAVs, is relevant. In our study the vaccine strains represent the most frequently detected influenza virus lineages (H1-1A.3.3.2; H1-1B.2.4; H3-1990.5.2) in pigs in Brazil [1, 3, 4]. As a significant antigenic variation exists between them, no cross-reactive antibody response in vaccinated pigs was expected.

The virosomal vaccines have been explored as an alternative to the conventional vaccine platforms, such as WIV vaccines, since they elicit both humoral and cellular immune responses while maintaining a favorable safety profile [19, 20]. Research on virosome vaccines, specifically in the context of targeting influenza or swine-related pathogens, remains relatively scarce. Virosome-based influenza vaccines have been reported for use in chickens [22] and humans [21]. Additionally, they have been investigated in mice [44] and ferrets [45] as an experimental model. This vaccine technology has also been successfully applied for other viruses infecting both animals, such as Newcastle disease virus in poultry [46] and avian metapneumovirus in turkeys [47], or humans, as seen with SARS-CoV-2 [48] and hepatitis A [49]. In this study, we demonstrated the immunogenicity in swine of a virosomal influenza vaccine containing the glycoproteins of the most prevalent virus subtypes circulating in Brazilian pig herds. Previously, the vaccine safety was evaluated in mice through the analysis of liver and kidney function, histopathology, and in vitro and in vivo cytotoxicity assays. It was demonstrated that the vaccine formulation was safe and non-cytotoxic for mice [24].

In swine, a robust humoral and cellular immune response was induced after two doses of the vaccine. HI and virus-neutralizing antibodies were detected against H1N1pdm, H1N2 and H3N2 viruses after booster immunization. In humans, the intramuscular administration of a virosomal influenza vaccine induced HI titers like those induced by WIV or subunit vaccines [50]. In poultry vaccinated by a virosomal influenza vaccine, low HI titers were detected seven days post vaccination, showing a significant rise in HI titers 14 days post vaccination, that were maintained at day 28 post vaccination [22]. Antibodies raised against HA are correlated with protection from clinical disease and are important to block the virus attachment and entry, preventing the virus infection [7]. Virus-neutralizing antibodies also have a vital function in preventing the binding of IAV to sialic acid receptors, the fusion process, and the release of newly formed viral particles [51]. A commercial virosomal vaccine for influenza in humans is available in several countries. This vaccine was considered highly effective by mimicking natural infection, and was immunogenic in both healthy and immunocompromised elderly, adults, and children [21].

The induction of influenza HA-specific antibody titers is affected by virus dose [52], and possibly by the virus subtype. Some studies have shown that pigs infected with H3N2 had higher antibody titers than those infected with H1N1 and H1N2 [53, 54]. Moreover, some viruses are more immunogenic than others, have dominant epitopes, and induce higher antibody titers [9]. The process of virus glycoprotein incorporation into the virosome is random, and as this formulation contained the glycoproteins from three different viruses, it is possible that a different concentration of each glycoprotein has been incorporated. According to a previously conducted SDS-PAGE, the virosome formulation used here contained more HA from the H3N2 virus, followed by H1N2 and H1N1 viruses [24]. This could explain high antibody titer for H3N2 virus compared to the antibody titers for H1N1 and H1N2 viruses by HI and SVN assays. Although antibody titers were not high for H1N1 and H1N2 subtypes, a robust cellular immune response was observed for these viruses.

In our study, immunization by a virosome-formulated influenza vaccine primed peripheral blood T and B cell subsets for early recall responses to H1N1pdm, H1N2 and H3N2 strains, including central memory CD4^+^ T cells (CD4^+^CD27^+^CD45RA^−^), cytotoxic T lymphocytes (CD3e^+^CD8^+^), and B lymphocytes (CD79a^+^, CD79a^+^SWC7^+^CD5^+^ and CD79a^+^SWC7^+^CD5^−^). As indicated, the virosome vaccine was optimal for stimulation of cell-mediated immunity, cytotoxic T lymphocyte (CTL) activity and in particular, central memory CD4^+^ T cells. CTL is responsible for elimination of virus-infected cells and clearance of influenza virus infection [55]. The central memory CD4^+^ T cells and B cells subsets (CD79a^+^, CD79a^+^SWC7^+^CD5^+^ and CD79a^+^SWC7^+^CD5^−^) are crucial for the development of memory B cells, antibody production, and antibody class switching [19]. Only the B cell subset CD79a^+^CD5^+^ and macrophages differed in BALF samples from vaccinated pigs, revealing a differentiated cellular response in the mucosa even though the vaccine has been administered by the intramuscular route.

After the in vitro stimulation of splenocytes from the vaccinated group, evidence of cellular immune response was marked by high maturation of macrophages, and high proliferation of B lymphocyte subsets (conventional B cells), T lymphocyte subsets (effector and central memory CD4^+^ and effector and central memory CD8^+^), and CD8^+^IFNγ^+^ T cells (CTL). The T cell subsets with the most significant responses to viral stimulation were the central memory helper (CD3e^+^CD4^+^CD27^+^) and cytotoxic (CD3e^+^CD8α^+^CD27^+^) T cells. These cell populations can mediate T helper function and express perforin to mediate cytolytic activity against virus infected cells [56, 57]. Taken together, these populations are required for enduring CD8^+^ T cell memory [58]. In addition, the virosome influenza vaccine induced significant proliferation of CD3e^+^CD8α^+^IFNγ^+^ T cells in swine, similar to previous studies in vaccinated mice [44], and pigs inoculated with a nanoparticle-adjuvanted influenza vaccine [59]. The whole inactivated virus (WIV) vaccine does not induce significant CTL activity, but the virosome is optimal for delivery of the antigen to the cytosol of antigen-presenting cells contributing to the clearance of influenza virus [55]. Nonetheless, higher levels of B cells (CD79a^+^SWC7^+^) proliferated in the splenocytes from the vaccinated pigs. These findings suggest that long-term memory cells preferentially traffic in lymphoid tissues like the spleen [60]. This could explain their low frequency in peripheral blood and BALF after immunization of pigs, in contrast to what was observed in the spleen following the in vitro stimulation. The characteristic recall responses of the two CD27^+^ populations, following the in vitro stimulation, became noticeably different, indicating that they were likely biased to a T helper 1 (Th1), T helper 2 (Th2) or T follicular helper (Tfh) phenotype. The commitment of memory CD4^+^ T cells to Th1 or Tfh lineages and memory CD8^+^ T cells to Th2 lineages provide cells that are poised for the lineage-specific expression of effector molecules upon preexposure to antigen. Upon recognizing virus-infected cells, CD8^+^ T cells readily respond by killing infected cells by producing antiviral cytokines and promoting the recruitment of immune cells [61]. These findings have important implications for vaccine design, as adjuvanted-virosomal vaccines could promote a higher quantity and quality of memory Tfh cells, potentially allowing for enhanced humoral immunity after prime and boost vaccination. In this way, specific cells generate responses against IAV that can help eliminate the virus from the infected cells. Moreover, they provide protection against future infections with the presence of memory CD4^+^ and CD8^+^ T cells that can respond quickly to new virus infections. This broad proliferation of different immune cells, acting in association as observed here, locally, and systemically, is important for the mitigation of influenza infection in swine herds.

Another relevant T-cell response data, after the in vitro stimulation, was that both CD3^+^CD4^+^ and CD3^+^CD8^+^ cells induced an upregulation in CD25 expression. It indicates cell activation [12], which might be associated with a protection response against IAV as observed in clinical challenge studies [62, 63]. These findings indicated that immunization of pigs by an influenza-virosomal vaccine efficiently primes and activates CD8^+^ T cells that are important for the elimination of virus-infected cells, and reduction of virus shedding [64]. It is not clear why the CD3e^+^CD4^+^CD25^+^ and CD3e^+^CD8α^+^CD25^+^ responses of pigs vaccinated with H1N1, H1N2 and H3N2 remained lower than CDe3^+^CD4^+^CD27^+^ and CD3e^+^CD8α^+^CD27^+^ after in vitro stimulation. One possibility is that the IAV antigens activated in vitro the quiescent memory cells, and the analysis time after the activation of memory cells was insufficient to activate and detect the effector cells.

For the design of IAV vaccines, both humoral and cellular immunity should be considered, along with the capability to elicit strong and long-lasting immunity. In the production system in Brazil, the pig market age is around 130–140 days. Here, we assessed the immune response in pigs 90 days after the first vaccine dose, when pigs reached 130 days old. Despite the antibody titers were low at 90 days after vaccination, high proliferation of B lymphocytes, as well as central memory CD4^+^ and CD8^+^ T lymphocytes, and CTL (CD3e^+^CD8α^+^IFNγ^+^) were demonstrated for the three vaccine viruses (H1N1pdm, H1N2 and H3N2) on day 90. Moreover, the specific memory cells detected 90 days’ post-vaccination allow rapid clonal expansion in a future exposure to IAV. Although a low number of pigs (n = 3) was evaluated, our results are encouraging since the immune response for influenza after vaccination persisted during the production phase when pigs are more at risk of influenza infection, during the nursery and the finishing phase. Further studies are needed to assess the duration of immunity induced by the virosome vaccine in swine.

## Conclusions

In conclusion, the virosomal-based influenza vaccine developed here showed a robust antibody- and cell-mediated immune responses in pigs, with the potential to confer long-lasting immune memory to pigs (until the market age), and proved to be safe. It also allows for the rapid update of vaccine virus components. Additional studies are required to assess whether the vaccine also induced NA-specific antibodies and to ascertain whether this vaccine formulation is protective against the in vivo challenge by swIAV. For vaccine composition, selection of swIAV strains, by antigenic cartography, that better match with circulating viruses in swine in Brazil may contribute to the control of influenza in swine herds, reducing virus transmission among pigs, and the potential likelihood of generation of novel viruses.

### Supplementary Information

**Additional file 1**: **Fig. S1.** TUNEL assay. Number of apoptotic cells observed in the TUNEL assay in different tissues (lung, mediastinal lymph node, spleen, liver and kidney) from pigs in the non-vaccinated (G1) and vaccinated (G2) groups on D28 postvaccination. Data are displayed for each pig per group and the black lines represent the mean ± standard deviation.

**Additional file 2**: **Fig. S2.** Humoral immune response. Antibody titers by **A** hemagglutination inhibition (HI) and **B** serum virus neutralization (SVN) assays for H1N1, H1N2 and H3N2 subtypes of serum samples collected from pigs in the vaccinated (G2) group on D60 and D90 post-vaccination. Data are shown for each pig and the black lines represent the geometric mean titers ± standard deviation.

**Additional file 3**: **Table S1**. Data from the in vitro cell proliferation assay. Fold change means and standard errors of immune cells in the splenocyte proliferation assay stimulated with the vaccine viruses (H1N1, H1N2 and H3N2), from the vaccinated group (G2) over the non-vaccinated group (G1) on D28 and D90 post-vaccination.

**Additional file 4**: **Fig. S3.** Cellular immune response. Immune cells in the **A** in vitro splenocyte proliferation assay stimulated with the vaccine viruses (H1N1, H1N2 and H3N2), **B** peripheral blood mononuclear cells (PBMCs), and C bronchoalveolar lavage fluid (BALF) cells, as a fold change from the vaccinated group (G2) over the non-vaccinated group (G1) on D90 post-vaccination. Data are shown for each pig and the black lines represent the mean ± standard error. Asterisks (*) denote significant differences between non-vaccinated (G1) and vaccinated (G2) groups (*P* ≤ 0.05).

## Data Availability

The authors confirm that all relevant data are included in the paper or in the supplementary material. Additional information is available from the authors on reasonable request.

## Abbreviations

AEC: 3-Amino-9-ethylcarbazole
BALF: Bronchoalveolar lavage fluid
BSA: Bovine serum albumin
BSL1: Biosafety level 1
CFSE: Carboxyfluorescein succinimidyl ester
CS&T: Cytometer Setup and Tracking
CTL: Cytotoxic T lymphocyte
D: Day
DCPC: 1,2-Dicaproyl-sn-glycero-3-phosphocholine
FBS: Fetal bovine serum
FMO: Fluorescence minus one
FSC: Forward scatter
FSC-A: Forward scatter area
FSC-H: Forward scatter height
HA: Hemagglutinin
H&E: Hematoxylin and eosin
HI: Hemagglutination inhibition
IAV: Influenza A virus
IFN: Interferon
LAIV: Live-attenuated influenza vaccine
mAbs: Monoclonal antibodies
MDCK: Madin–Darby canine kidney
MHC: Major histocompatibility complex
NA: Neuraminidase
NK: Natural killer
PBMC: Peripheral blood mononuclear cell
PCV2: Porcine circovirus type 2
Pdm: Pandemic
PRRSV: Porcine reproductive and respiratory syndrome virus
SAS: Statistical analysis system
SPF: Specific pathogen-free
SSC: Side scatter
SVN: Serum virus neutralization
swIAV: Swine influenza A virus
Tfh: T follicular helper
Th: T helper
TNE: Tris-NaCl-EDTA
TUNEL: Terminal deoxynucleotidyl transferase-mediated deoxyuridine triphosphate nick-end labeling
WIV: Whole inactivated virus

## References

[1] Anderson TK, Chang J, Arendsee ZW, Venkatesh D, Souza CK, Kimble JB (2021). Swine influenza A viruses and the tangled relationship with humans. Cold Spring Harb Perspect Med.

[2] Nelson MI, Schaefer R, Gava D, Cantão ME, Ciacci-Zanella JR (2015). Influenza A viruses of human origin in swine. Brazil Emerg Infect Dis.

[3] Tochetto C, Junqueira DM, Anderson TK, Gava D, Haach V, Cantão ME (2023). Introductions of human-origin seasonal H3N2, H1N2 and pre-2009 H1N1 influenza viruses to swine in Brazil. Viruses.

[4] Junqueira DM, Tochetto C, Anderson TK, Gava D, Haach V, Cantão ME, Baker ALV, Schaefer R (2023). Human-to-swine introductions and onward transmission of 2009 H1N1 pandemic influenza viruses in Brazil. Front Microbiol.

[5] Schaefer R, Lopes S, Gava D, Ciacci-Zanella JR, Anderson TK, Lewis NS, et al. Genetic and antigenic diversity of contemporary influenza A virus in swine in Brazil. Proc Int Pig Vet Soc Congr. Rio de Janeiro; 2020. p. 828.

[6] Rajão DS, Walia RR, Campbell B, Gauger PC, Janas-Martindale A, Killian ML (2017). Reassortment between swine H3N2 and 2009 pandemic H1N1 in the United States resulted in influenza A viruses with diverse genetic constellations with variable virulence in pigs. J Virol.

[7] Krammer F (2019). The human antibody response to influenza A virus infection and vaccination. Nat Rev Immunol.

[8] Koel BF, Mögling R, Chutinimitkul S, Fraaij PL, Burke DF, van der Vliet S (2015). Identification of amino acid substitutions supporting antigenic change of influenza A(H1N1)pdm09 viruses. J Virol.

[9] Vincent AL, Perez DR, Rajao D, Anderson TK, Abente EJ, Walia RR (2017). Influenza A virus vaccines for swine. Vet Microbiol.

[10] Mancera Gracia JC, Pearce DS, Masic A, Balasch M (2020). Influenza A virus in swine: epidemiology, challenges and vaccination strategies. Front Vet Sci.

[11] Van Reeth K, Ma W (2013). Swine influenza virus vaccines: to change or not to change—that’s the question. Curr Top Microbiol Immunol.

[12] Kappes MA, Sandbulte MR, Platt R, Wang C, Lager KM, Henningson JN (2012). Vaccination with NS1-truncated H3N2 swine influenza virus primes T cells and confers cross-protection against an H1N1 heterosubtypic challenge in pigs. Vaccine.

[13] Sharma A, Zeller MA, Li G, Harmon KM, Zhang J, Hoang H (2020). Detection of live attenuated influenza vaccine virus and evidence of reassortment in the U.S. swine population. J Vet Diagnostic Investig..

[14] Braucher DR, Henningson JN, Loving CL, Vincen AL, Kim E, Steitz J (2012). Intranasal vaccination with replication-defective adenovirus type 5 encoding influenza virus hemagglutinin elicits protective immunity to homologous challenge and partial protection to heterologous challenge in pigs. Clin Vaccine Immunol.

[15] Joshi LR, Knudsen D, Piñeyro P, Dhakal S, Renukaradhya GJ, Diel DG (2021). Protective efficacy of an orf virus-vector encoding the hemagglutinin and the nucleoprotein of influenza A virus in swine. Front Immunol.

[16] Borggren M, Nielsen J, Karlsson I, Dalgaard TS, Trebbien R, Williams JA (2016). A polyvalent influenza DNA vaccine applied by needle-free intradermal delivery induces cross-reactive humoral and cellular immune responses in pigs. Vaccine.

[17] Hernandez LA, Miller CL, Vaughn EM (2016). Particle and subunit-based hemagglutinin vaccines provide protective efficacy against H1N1 influenza in pigs. Vet Microbiol.

[18] Soema PC, Kompier R, Amorij JP, Kersten GFA (2015). Current and next generation influenza vaccines: formulation and production strategies. Eur J Pharm Biopharm.

[19] Huckriede A, Bungener L, Stegmann T, Daemen T, Medema J, Palache AM (2005). The virosome concept for influenza vaccines. Vaccine.

[20] Wilschut J (2009). Influenza vaccines: the virosome concept. Immunol Lett.

[21] Herzog C, Hartmann K, Künzi V, Kürsteiner O, Mischler R, Lazar H (2009). Eleven years of Inflexal® V-a virosomal adjuvanted influenza vaccine. Vaccine.

[22] Mallick AI, Parvizi P, Read LR, Nagy É, Behboudi S, Sharif S (2011). Enhancement of immunogenicity of a virosome-based avian influenza vaccine in chickens by incorporating CpG-ODN. Vaccine.

[23] Zhang J, Gauger PC (2020). Isolation of swine influenza A virus in cell cultures and embryonated chicken eggs. Methods Mol Biol.

[24] Fonseca F, Haach V, Bellaver F, Bombassaro G, Gava D, Paulino L, et al. Immunological profile of mice immunized with a polyvalent virosome-based influenza vaccine. Preprint at 10.21203/rs.3.rs-2923914/v1 (2023). 10.1186/s12985-023-02158-0 PMC10463652 37605141

[25] Zhang J, Harmon KM (2020). RNA extraction from swine samples and detection of influenza A virus in swine by real-time RT-PCR. Methods Mol Biol.

[26] Lager KM, Vincent AL (2020). In vivo models for pathotyping and vaccine efficacy for swine influenza. Methods Mol Biol.

[27] Opriessnig T, Yu S, Gallup JM, Evans RB, Fenaux M, Pallares F (2003). Effect of vaccination with selective bacterins on conventional pigs infected with type 2 porcine circovirus. Vet Pathol.

[28] Schaller A, Djordjevic SP, Eamens GJ, Forbes WA, Kuhn R, Kuhnert P (2001). Identification and detection of Actinobacillus pleuropneumoniae by PCR based on the gene apxIVA. Vet Microbiol.

[29] Turni C, Pyke M, Blackall PJ (2010). Validation of a real-time PCR for Haemophilus parasuis. J Appl Microbiol.

[30] Dubosson CR, Conzelmann C, Miserez R, Boerlin P, Frey J, Zimmermann W (2004). Development of two real-time PCR assays for the detection of *Mycoplasma hyopneumoniae* in clinical samples. Vet Microbiol.

[31] Townsend KM, Boyce JD, Chung JY, Frost AJ, Adler B (2001). Genetic organization of *Pasteurella multocida cap* loci and development of a multiplex capsular PCR typing system. J Clin Microbiol.

[32] Gava D, Caron L, Schaefer R, Silva VS, Weiblen R, Flores EF (2022). A retrospective study of porcine reproductive and respiratory syndrome virus infection in Brazilian pigs from 2008 to 2020. Transbound Emerg Dis.

[33] Prophet EB, Mills B, Arrington JB, Sobin LH (1992). Laboratory Methods in Histotechnology.

[34] Gava D, Zanella EL, Morés N, Ciacci-Zanella JR (2008). Transmission of porcine circovirus 2 (PCV2) by semen and viral distribution in different piglet tissues. Pesqui Veterinária Bras.

[35] Kitikoon P, Gauger PC, Vincent AL (2014). Hemagglutinin inhibition assay with swine sera. Methods Mol Biol.

[36] Gauger PC, Vincent AL (2014). Serum virus neutralization assay for detection and quantitation of serum-neutralizing antibodies to influenza A virus in swine. Methods Mol Biol.

[37] Dawson HD, Lunney JK (2018). Porcine cluster of differentiation (CD) markers 2018 update. Res Vet Sci.

[38] Maciag SS, Bellaver FV, Bombassaro G, Haach V, Morés MAZ, Baron LF (2022). On the influence of the source of porcine colostrum in the development of early immune ontogeny in piglets. Sci Rep.

[39] SAS. System for Microsoft Windows. Cary, NC, USA; 2012.

[40] Rajão DS, Costa ATR, Brasil BSAF, Del Puerto HL, Oliveira FG, Alves F (2013). Genetic characterization of influenza virus circulating in Brazilian pigs during 2009 and 2010 reveals a high prevalence of the pandemic H1N1 subtype. Influenza Other Respi Viruses.

[41] Schaefer R, Rech RR, Gava D, Cantão ME, da Silva MC, Silveira S (2015). A human-like H1N2 influenza virus detected during an outbreak of acute respiratory disease in swine in Brazil. Arch Virol.

[42] Resende PC, Born PS, Matos AR, Motta FC, Caetano BC, Debur M do C, et al. Whole-genome characterization of a novel human influenza A(H1N2) virus variant, Brazil. Emerg Infect Dis. 2017;23:152–4. 10.3201/eid2301.161122 PMC5176240 27983507

[43] Schaefer R, Zanella JRC, Brentano L, Vincen AL, Ritterbusc GA, Silveira S, et al. Isolation and characterization of a pandemic H1N1 influenza virus in pigs in Brazil. Pesqui Veterinária Bras. 2011;31.

[44] Kammer AR, Amacker M, Rasi S, Westerfeld N, Gremion C, Neuhaus D (2007). A new and versatile virosomal antigen delivery system to induce cellular and humoral immune responses. Vaccine.

[45] Lambkin R, Oxford JS, Bossuyt S, Mann A, Metcalfe IC, Herzog C (2004). Strong local and systemic protective immunity induced in the ferret model by an intranasal virosome-formulated influenza subunit vaccine. Vaccine.

[46] Rasool MH, Mehmood A, Saqalein M, Nisar MA, Almatroudi A, Khurshid M. Development, biological characterization, and immunological evaluation of virosome vaccine against Newcastle disease in Pakistan. Biomed Res Int. 2021. 10.1155/2021/8879277 PMC7864732 33575353

[47] Kapczynski DR (2004). Development of a virosome vaccine against avian metapneumovirus subtype C for protection in turkeys. Avian Dis.

[48] van der Velden YU, Grobben M, Caniels TG, Burger JA, Poniman M, Oomen M (2022). A SARS-CoV-2 Wuhan spike virosome vaccine induces superior neutralization breadth compared to one using the Beta spike. Sci Rep.

[49] Van Der Wielen M, Vertruyen A, Froesner G, Ibáñez R, Hunt M, Herzog C (2007). Immunogenicity and safety of a pediatric dose of a virosome-adjuvanted hepatitis A vaccine: a controlled trial in children aged 1–16 years. Pediatr Infect Dis J.

[50] Glück R, Mischler R, Finkel B, Que JU, Cryz SJ, Scarpa B (1994). Immunogenicity of new virosome influenza vaccine in elderly people. Lancet.

[51] Sicca F, Neppelenbroek S, Huckriede A (2018). Effector mechanisms of influenza-specific antibodies: neutralization and beyond. Expert Rev Vaccines.

[52] Waffarn EE, Baumgarth N (2011). Protective B cell responses to flu—no fluke!. J Immunol.

[53] Van Reeth K, Labarque G, Pensaert M (2006). Serological profiles after consecutive experimental infections of pigs with European H1N1, H3N2, and H1N2 swine influenza viruses. Viral Immunol.

[54] Van Reeth K, Gregory V, Hay A, Pensaert M (2003). Protection against a European H1N2 swine influenza virus in pigs previously infected with H1N1 and/or H3N2 subtypes. Vaccine.

[55] Schmidt ME, Varga SM (2018). The CD8 T cell response to respiratory virus infections. Front Immunol..

[56] De Bruin TGM, Van Rooij EMA, De Visser YE, Bianchi ATJ (2000). Cytolytic function for pseudorabies virus-stimulated porcine CD4+ CD8dull+ lymphocytes. Viral Immunol.

[57] Denyer MS, Wileman TE, Stirling CMA, Zuber B, Takamatsu HH (2006). Perforin expression can define CD8 positive lymphocyte subsets in pigs allowing phenotypic and functional analysis of natural killer, cytotoxic T, natural killer T and MHC un-restricted cytotoxic T-cells. Vet Immunol Immunopathol.

[58] Cullen JG, McQuilten HA, Quinn KM, Olshansky M, Russ BE, Morey A (2019). CD4+ T help promotes influenza virus-specific CD8+ T cell memory by limiting metabolic dysfunction. Proc Natl Acad Sci U S A.

[59] Dhakal S, Lu F, Ghimire S, Renu S, Lakshmanappa YS, Hogshead BT (2019). Corn-derived alpha-D-glucan nanoparticles as adjuvant for intramuscular and intranasal immunization in pigs. Nanomed Nanotechnol Biol Med.

[60] Hale JS, Youngblood B, Latner DR, Mohammed AUR, Ye L, Akondy RS (2013). Distinct memory CD4+ T cells with commitment to T follicular helper- and T helper 1-cell lineages are generated after acute viral infection. Immunity.

[61] Mackay LK, Stock AT, Ma JZ, Jones CM, Kent SJ, Mueller SN (2012). Long-lived epithelial immunity by tissue-resident memory T (TRM) cells in the absence of persisting local antigen presentation. Proc Natl Acad Sci U S A.

[62] Sridhar S, Begom S, Bermingham A, Hoschler K, Adamson W, Carman W (2013). Cellular immune correlates of protection against symptomatic pandemic influenza. Nat Med.

[63] Wilkinson TM, Li CKF, Chui CSC, Huang AKY, Perkins M, Liebner JC (2012). Preexisting influenza-specific CD4+ T cells correlate with disease protection against influenza challenge in humans. Nat Med.

[64] Thomas PG, Keating R, Hulse-Post DJ, Doherty PC (2006). Cell-mediated protection in influenza infection. Emerg Infect Dis.

