# Supplementary figures and images for: A polyvalent virosomal influenza vaccine induces broad cellular and humoral immunity in pigs
Source: Virol J. 2023 Aug 16;20:181. doi: 10.1186/s12985-023-02153-5 (PMC10428566; doi:10.1186/s12985-023-02153-5)

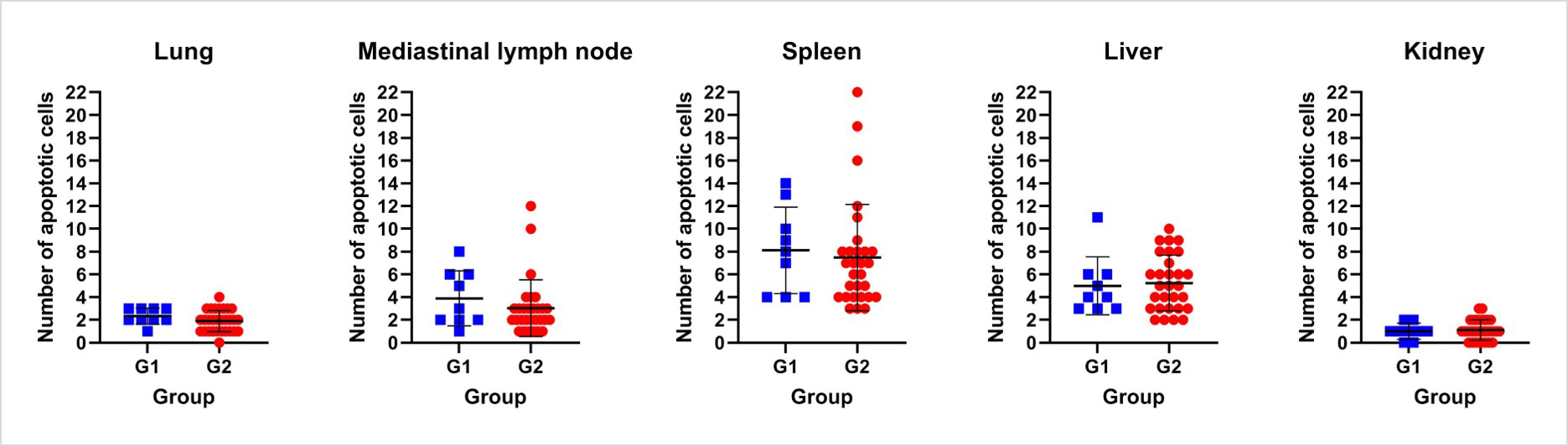

Supplement: Supplementary file 1 — Additional file 1: Fig. S1. TUNEL assay. Number of apoptotic cells observed in the TUNEL assay in different tissues (lung, mediastinal lymph node, spleen, liver and kidney) from pigs in the non-vaccinated (G1) and vaccinated (G2) groups on D28 postvaccination. Data are displayed for each pig per group and the black lines represent the mean ± standard deviation. [file 12985_2023_2153_MOESM1_ESM.jpg]

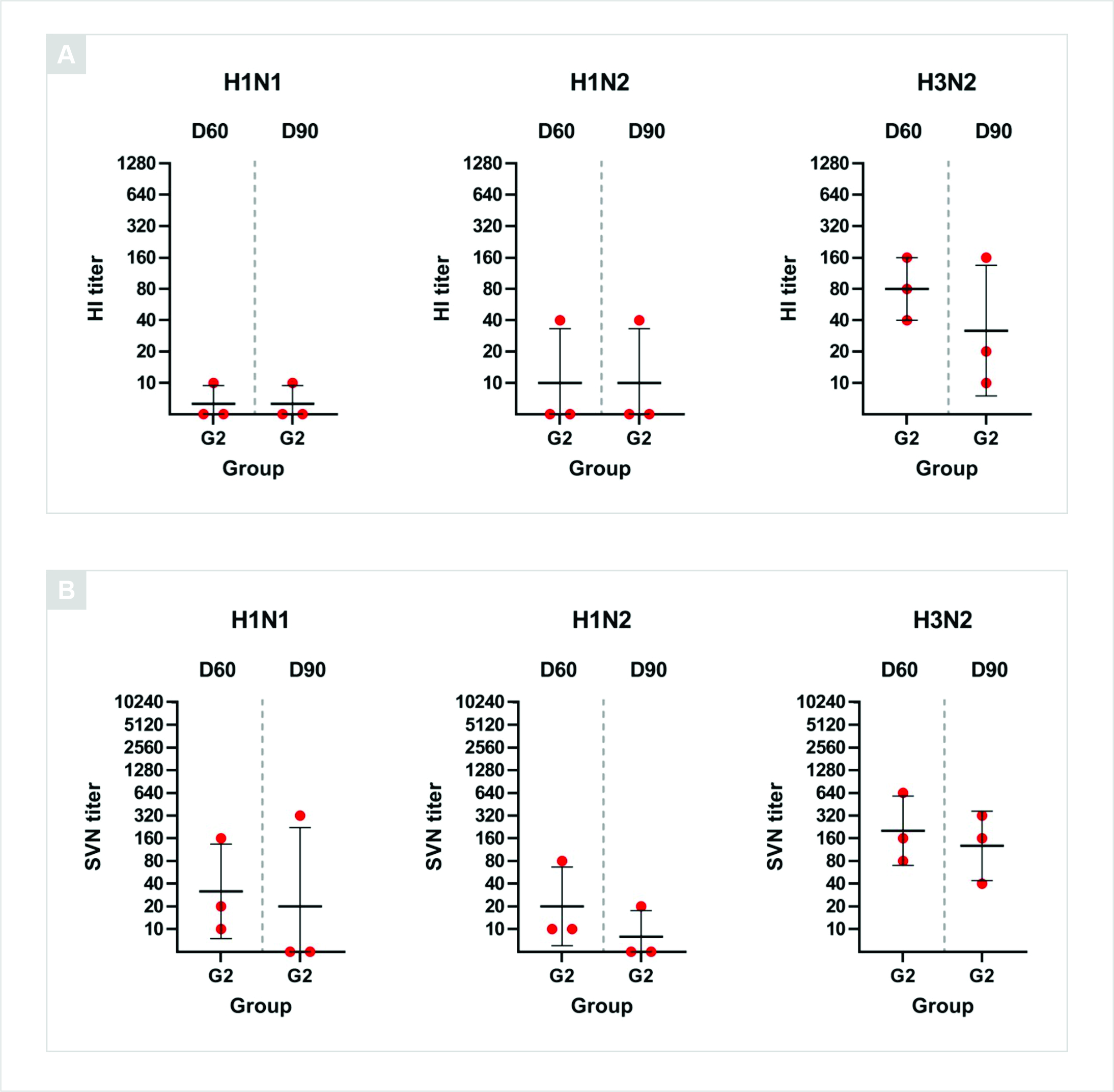

Supplement: Supplementary file 2 — Additional file 2: Fig. S2. Humoral immune response. Antibody titers by A hemagglutination inhibition (HI) and B serum virus neutralization (SVN) assays for H1N1, H1N2 and H3N2 subtypes of serum samples collected from pigs in the vaccinated (G2) group on D60 and D90 post-vaccination. Data are shown for each pig and the black lines represent the geometric mean titers ± standard deviation. [file 12985_2023_2153_MOESM2_ESM.jpg]

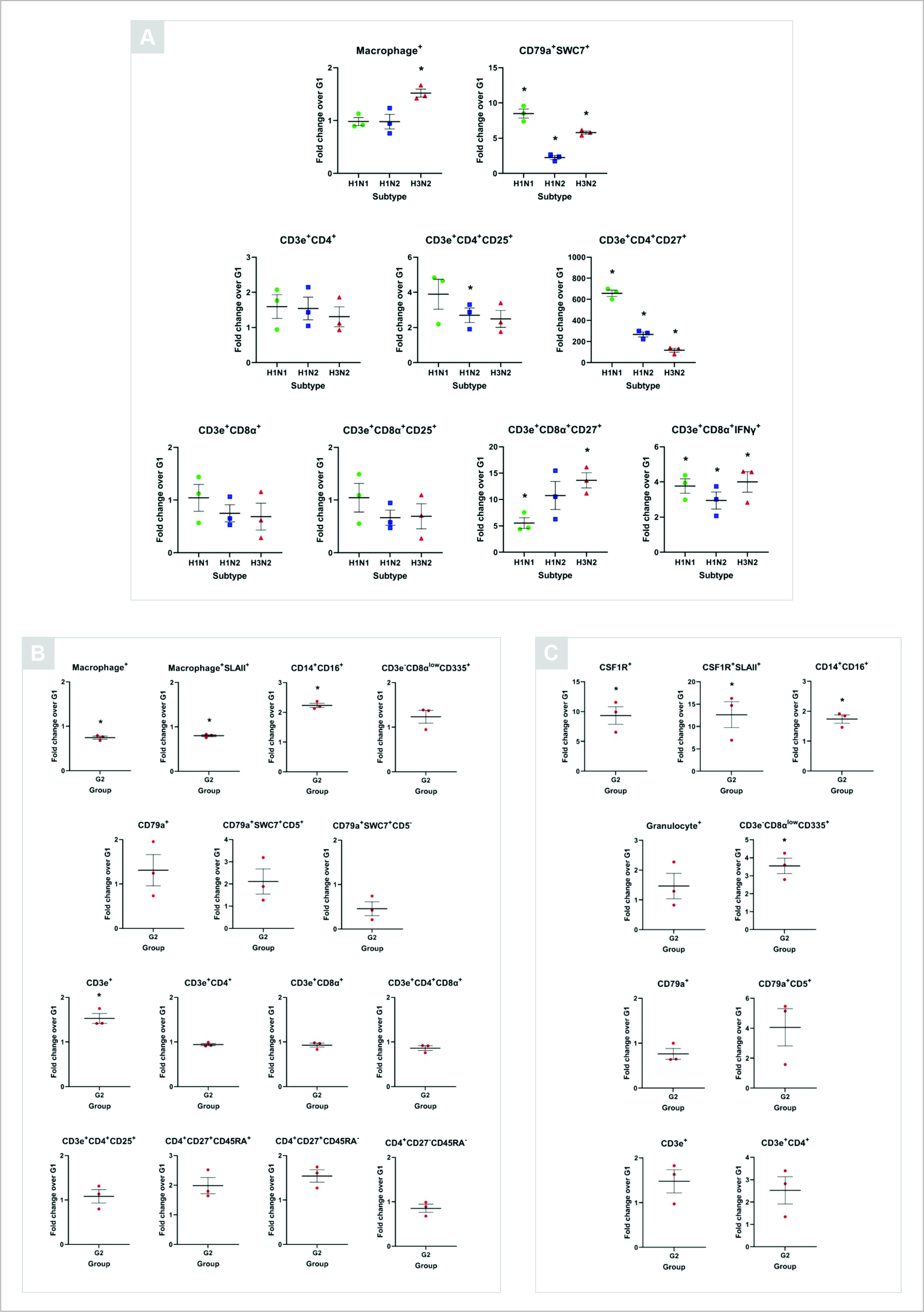

Supplement: Supplementary file 4 — Additional file 4: Fig. S3. Cellular immune response. Immune cells in the A in vitro splenocyte proliferation assay stimulated with the vaccine viruses (H1N1, H1N2 and H3N2), B peripheral blood mononuclear cells (PBMCs), and C bronchoalveolar lavage fluid (BALF) cells, as a fold change from the vaccinated group (G2) over the non-vaccinated group (G1) on D90 post-vaccination. Data are shown for each pig and the black lines represent the mean ± standard error. Asterisks (*) denote significant differences between non-vaccinated (G1) and vaccinated (G2) groups (P ≤ 0.05). [file 12985_2023_2153_MOESM4_ESM.jpg]
