# Supplementary material for: A polyvalent virosomal influenza vaccine induces broad cellular and humoral immunity in pigs
Source: Virol J. 2023 Aug 16;20:181. doi: 10.1186/s12985-023-02153-5 (PMC10428566; doi:10.1186/s12985-023-02153-5)
Supplement: Supplementary file 3 — Additional file 3: Table S1. Data from the in vitro cell proliferation assay. Fold change means and standard errors of immune cells in the splenocyte proliferation assay stimulated with the vaccine viruses (H1N1, H1N2 and H3N2), from the vaccinated group (G2) over the non-vaccinated group (G1) on D28 and D90 post-vaccination. [file 12985_2023_2153_MOESM3_ESM.docx]

**Table S1.** Data from the *in vitro* cell proliferation assay. Fold change means and standard errors of immune cells in the splenocyte proliferation assay stimulated with the vaccine viruses (H1N1, H1N2 and H3N2), from the vaccinated group (G2) over the non-vaccinated group (G1) on D28 and D90 post-vaccination.

| **Immune cells** | **D28** | | | **D90** | | |
| --- | --- | --- | --- | --- | --- | --- |
|  | **H1N1** | **H1N2** | **H3N2** | **H1N1** | **H1N2** | **H3N2** |
| Macrophage^+^  (Myeloid cells) | 1.49±0.10 (*P*<0.0001)* | 1.35±0.10 (*P*=0.002)* | 1.83±0.14 (*P*<0.0001)* | 0.98±0.07 (*P*=0.81) | 0.98±0.14 (*P*=0.89) | 1.52±0.07 (*P*=0.02)* |
| CD79a^+^SWC7^+^  (B cells) | 9.95±0.64 (*P*<0.0001)* | 3.05±0.21 (*P*<0.0001)* | 7.42±0.46 (*P*<0.0001)* | 8.49±0.64 (*P*=0.007)* | 2.26±0.28 (*P*=0.04)* | 5.79±0.22 (*P*=0.002)* |
| CD3e^+^CD4^+^  (CD4^+^ T cells) | 1.94±0.12 (*P*<0.0001)* | 1.94±0.10 (*P*<0.0001)* | 1.97±0.11 (*P*<0.0001)* | 1.59±0.34 (*P*=0.22) | 1.54±0.32 (*P*=0.23) | 1.31±0.28 (*P*=0.39) |
| CD3e^+^CD4^+^CD25^+^  (Effector CD4^+^ T cells) | 5.19±0.38 (*P*<0.0001)* | 4.03±0.30 (*P*<0.0001)* | 4.18±0.27 (*P*<0.0001)* | 3.89±0.85 (*P*=0.07) | 2.69±0.41 (*P*=0.05)* | 2.49±0.48 (*P*=0.09) |
| CD3e^+^CD4^+^CD27^+^  (Central memory CD4^+^ T cells) | 733.2±83.93 (*P*<0.0001)* | 319.2±40.19 (*P*<0.0001)* | 176.0±19.72 (*P*<0.0001)* | 657.1±29.74 (*P*=0.002)* | 267.3±23.39 (*P*=0.007)* | 117.7±18.72 (*P*=0.02)* |
| CD3e^+^CD8α^+^  (CD8^+^ T cells) | 1.39±0.09 (*P*=0.0001)* | 1.16±0.07 (*P*=0.03)* | 1.44±0.10 (*P*=0.0001)* | 1.04±0.25 (*P*=0.88) | 0.75±0.16 (*P*=0.26) | 0.69±0.25 (*P*=0.34) |
| CD3e^+^CD8α^+^CD25^+^  (Effector CD8^+^ T cells) | 2.09±0.19 (*P*<0.0001)* | 1.71±0.15 (*P*<0.0001)* | 2.14±0.21 (*P*<0.0001)* | 1.04±0.27 (*P*=0.88) | 0.67±0.14 (*P*=0.14) | 0.69±0.24 (*P*=0.32) |
| CD3e^+^CD8α^+^CD27^+^  (Central memory CD8^+^ T cells) | 8.88±0.73 (*P*<0.0001)* | 18.58±1.66 (*P*<0.0001)* | 19.26±1.57 (*P*<0.0001)* | 5.52±1.02 (*P*=0.04)* | 10.77±2.66 (*P*=0.06) | 13.64±1.43 (*P*=0.01)* |
| CD3e^+^CD8α^+^IFNγ^+^  (CD8^+^ T cells producing IFN-γ) | 3.59±0.18 (*P*<0.0001)* | 2.70±0.12 (*P*<0.0001)* | 3.72±0.17 (*P*<0.0001)* | 3.77±0.41 (*P*=0.02)* | 2.94±0.48 (*P*=0.05)* | 4.00±0.58 (*P*=0.03)* |

Asterisks (*) denote significant differences among non-vaccinated (G1) and vaccinated (G2) groups (*P*≤0.05).
